# Regenerative Endodontic Procedure in an Immature Permanent Incisor with Internal Root Resorption: a Case Report

**DOI:** 10.30476/DENTJODS.2022.88349.1328

**Published:** 2022-06

**Authors:** Gaizka Loroño, Antonio Jesús Conde, Roberto Estévez, Claudia Brizuela, Rafael Cisneros, Ruth Pérez Alfayate

**Affiliations:** 1 Postgraduate Program in Endodontics, European University of Madrid, Madrid, Spain; 2 Research Center in Oral Biology and Regeneration, Faculty of Dentistry, Universidad de Los Andes, Santiago, Chile

**Keywords:** Tooth intrusion, Odontogenesis, Root resorption, Regenerative endodontics, Mineral trioxide aggregate

## Abstract

This report describes a regenerative endodontic procedure of an immature permanent incisor with internal root resorption (IRR) and 4-years follow-up. A healthy 8-year-old man was referred for treatment of tooth #9 after a traumatic intrusion. The periapical radiograph showed an IRR and an open apex with periradicular lesion. A diagnosis of pulp necrosis and chronic apical abscess was achieved. In the first appointment, under local anesthesia and rubber dam isolation, an access cavity was designed and the root canal was chemically cleaned under irrigation with 10 mL 1.5% sodium hypochlorite (NaOCl). The root canal was then dried and calcium hydroxide paste was placed. During the second appointment, the root canal was irrigated with 5 mL of 17% ethylenediaminetetraacetic acid (EDTA) for 5 minutes and dried. The blood clot was established in a time of 3 minutes after the bleeding from the periapical tissue was trigged. White mineral trioxide aggregate (MTA) was placed up to the amelocemental junction and the final restoration of the access cavity was carried out. During periodic clinical and radiographic follow-up, the patient remained symptom free, the periapical region was completely healed, inhibition of the root resorption process achieved, and formation of the new periodontal ligament as well as tooth widening development observed, meeting functional expectations after 48 months. The regenerative endodontic procedures are an available option to treat IRR in severely immature teeth. The available literature on the regenerative endodontic procedures applied to IRR treatment is limited, and more research is needed in this field.

## Introduction

Root resorption occurs due to the loss of dental hard tissues as a result of the action of odontoclasts [ 1 ] and might be classified into external or internal resorption depending on the location of the lesion in relation to the root surface [ 2 ]. Internal root resorption (IRR), being the less commonly occurring type of root resorption [ 3 ], is a resorptive defect of the internal aspect of the root [ 4 ]. The image of this lesion is normally round to oval and continues within the canal space.

Its pathogenesis occurs because of the activity of odontoclasts, which are multinucleated cells able to form

resorption lacunae [ 4 ]. For these cells can perform their function, thus, for IRR to occur, the protective odontoblasts layer and predentine surrounding the canal wall should be pre-damaged originating the exposure of the underlying mineralized dentin to odontoclasts [ 5 ]. Without bacterial stimulation, the resorption will be self-limited. However, if there is a chronic stimulation through the infected necrotic coronal tissue, and a viable blood supply from the pulp tissue apical to the resorptive lesion, those clastic cells will continue in time with their function [ 2 ], being able to produce a perforation with the periodontal ligament [ 6 ], worsening the prognosis and hindering the treatment. Traditionally, due to the idiosyncrasy of IRR, root canal treatment has been the treatment of choice and requires chemo-mechanical preparation and root canal obturation with thermoplastic gutta-percha techniques [ 3 ].

In some cases, a surgical approach is indicated, predominantly when the area of resorption cannot be accessed through the canal or has progressed through the tooth and has reached the periodontium [ 7 ].

The foundation of “revascularization” and “regenerative endodontic treatment” (RET) terms was started in the 60´s [ 8 ]. RET allows the regeneration of the root by reestablishing blood flow to the tooth in cases of pulp necrosis in permanent teeth with open apex, not only eliminating and/or preventing apical periodontitis, but the promotion of root development, and hence preventing root fracture [ 9 ]. The most recent innovation in the treatment of IRR is RET since this option provides the opportunity for replacement of missing structure caused by the resorption and thus, giving a better prognosis in the long-term for those teeth affected [ 4 , 10 ]. Nevertheless, RET for IRR is a technique that, to our knowledge, has only be applied in teeth with completed developed roots. Therefore, the aim of this case report is to present a RET procedure of a perforated IRR in a tooth with open apex and its clinical and radiographic findings after 48 months.

## Case Presentation

**Figure 1:**
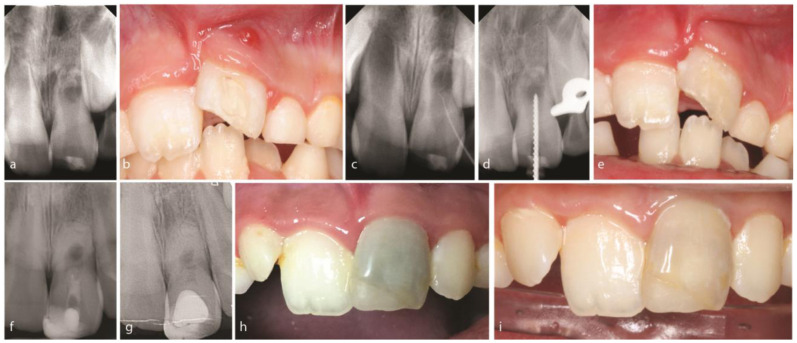
**a:** The initial radiograph taken 5 days before first visit shows internal root resorption and an open apex, **b:** Sinus tract at teeth #9, **c:** radiograph with a gutta-percha cone through the sinus tract, **d:** working length with a #80 K-file, **e:** absence of sinus tract in the second appointment, **f:** The 24 month radiograph shows a complete healing of the periradicular lesion, inhibition of the root resorption process, and formation of the new periodontal ligament as well as tooth widening development, **g:** a favorable evolution of the treatment at 48 months with development of a hard tissue bridge and MTA removal, **h:** photography that shows discoloration of the tooth, **i:** whitening of the tooth after MTA removal, and restoration with resin composite and a buccal direct composite veneer.

An 8-year-old Caucasian healthy male was referred for the management of tooth #9 after a traumatic injury in a swimming pool occurred 3 months before. The dental history from the hospital and the clinic that the patient visited the first time revealed an intrusion-injury as well as a severe luxation in which the longitudinal axis of the tooth was positioned perpendicular to its initial position, cortical bone fracture and mobility of all anterosuperior teeth that have been treated before at the hospital (Figure 1a).

To endodontic inspection and exploration an uncomplicated crown fracture of tooth #9 has been observed. Tooth #9 was sensitive to palpation and percussion, without mobility, and did not respond to cold and electric pulp sensitivity tests. A sinus tract at this level was also observed (Figure 1b) and a new radiograph was taken (Figure 1c). On the periapical radiograph it could be observed that tooth #9 was the cause for the sinus tract. An IRR and an open apex were also noted and confirmed by different radiographic projections. A diagnosis of pulp necrosis and chronic apical abscess with IRR was achieved. In order to obtain more information of the characteristics and dimensions of the resorption area, a cone beam computed tomography (CBCT) was indicated. Nevertheless, this was rejected for the patient´s tutor due to economic reasons and after the explanation of all the therapeutic options, RET was chosen and informed consent obtained.

Local anesthesia with 4% articaine containing 1:200,000 epinephrine (Ubistesine 4%; 3M ESPE AG, Seefeld, Germany) was performed, followed by rubber dam (Nic Tone, MD Dental, Zapopan, México), isolation. Access cavity was accomplished using a diamond bur (number 856, Komet Dental GmBH, Lemgo, Germany) and an Endo-access bur (Dentsply Maillefer, Tulsa, OK). Working length was established at 11 mm by using an electronic apex locator (Root ZX II, J. Morita Corp, Kyoto, Japan) and confirmed by a working length radiograph with a #80 K-File (Dentsply Maillefer, Tulsa, OK) (Figure 1d). Instrumentation was not carried out, but a gently and careful irrigation with 20 mL 1.5% sodium hypochlorite (NaOCl) was realized [ 11 ]. Subsequently the canal was irrigated with physiological saline in order to minimize the cytotoxic effect of NaOCl, dried with sterile paper points and irrigated with 20 mL of 17% ethylenediaminetetraacetic acid (EDTA) (Dentaflux, Madrid, Spain). Then, the canal was dried with sterile paper points and calcium hydroxide (Octocanal, Clarben, Madrid, Spain) was placed as interappointment medication [ 12 ]. The access was sealed with sterile cotton pellet and a temporary restoration with Cavit G (3M ESPE AG).

The patient was recalled 3 weeks later. The tooth was asymptomatic and sinus tract was not present (Figure 1e). After local anesthesia without a vasoconstrictor (mepivacaine 3%; 3M ESPE AG) and tooth isolation with rubber dam (Nic Tone, MD Dental, Zapopan, México), the canal was again accessed and irrigated with 20mL of 17% EDTA (Dentaflux) during 5 minutes in order to enhance the elimination of the calcium hydroxide and to open and clean the dentinal tubules.

The canal was then dried with sterile paper points. Bleeding was induced thought the use of#30 K-File (Dentsply Maillefer, Ballaigues, Switzerland) 3 mm beyond the apex. The blood clot was established in 3 minutes. A collagen sponge (Hemocollagene, Septodont, Saint-Maur-des-Fossés, France) was then placed in the coronal third of the canal to facilitate the placement of white mineral trioxide aggregate (MTA) (ProRoot MTA; Dentsply Maillefer, Ballaigues, Switzerland). As the working length was 11mm, it was a need to settle the MTA plug up to the cementoenamel junction. The access cavity was again sealed with sterile cotton pellet and filled with a temporary restoration (Cavit G, 3M ESPE AG). Three days later, MTA setting was confirmed and the cavity was sealed permanently with glass ionomer cement and light-cured composite resin.

Follow-up at 3, 6, 12, 24 and 48 months were achieved. Patient was asymptomatic, with physiological mobility and without sinus tract at all them. Periapical radiograph at 24 months (Figure 1f) showed already signs of complete healing of the periradicular lesion, inhibition of the root resorption process, and formation of the new periodontal ligament as well as tooth widening development. Non-improvement regarding root length was observed. Clinically, the tooth remained asymptomatic and functional, and the soft tissues maintained a physiological clinical appearance.

At 48 months, periapical radiograph revealed a favorable evolution of the treatment (Figure 1g). Discoloration of the tooth required at this moment a solution (Figure 1h). A gradual development of a hard tissue bridge was radiographically evident below the mass of MTA, and after the assessment of the situation, the removal of MTA and the placement of an aesthetic buccal composite restoration was proposed (Figure 1g). Thus, after local anesthesia and rubber dam isolation, the complete elimination of MTA was performed using a diamond bur (number 856, Komet Dental GmBH) under cooling water and operating microscope (OPMI pico Dental Microscope, Carl Zeiss, Oberkochen, Germany) until hard-tissue bridge was exposed. The access cavity was subsequently restored with resin composite and a buccal direct composite veneer. Whitening of the tooth was immediately evidenced (Figure 1i). The patient was recalled at 49, 51 and 54 months from the first visit with no color change of the crown. At the same time, it was recommended to consult an orthodontist.

## Discussion

The management and treatment of a traumatic injury to teeth that have not yet completed their root maturation are a challenge for the clinician, and even more if they develop a concomitant IRR. To our knowledge, there is a few papers which describe the treatment of IRR with RET [ 4 , 10 ] and there is lack of publications related to the treatment of IRR with RET in open apex.

Traumatic injuries have been described as the first cause of IRR, since it can generate a damage of the odontoblasts and predentine protective layer [ 7 ]. At the same time, as described previously [ 13 ] the traumatic injury to the incisor and its intrusion generates a damage to the periodontal ligament responsible of an uncertain and unfavorable environment for periradicular tissues that affects the normal root development, resulting in a short root with thin walls and open apex. 

Traditionally, the treatment of choice for teeth with pulp necrosis and open apex has been apexification [ 14 ]. On the other hand, the classical treatment for IRR has been conventional root canal treatment [ 3 ]. Whereas traditional root canal treatment is aimed to seal the root canal system with biocompatible materials and the objective of preventing reinfection, RET is intended to re-establish vitality, immunity and if possible, sensitivity of the pulp tissue. This is an important issue as only vital tissue has the capacity to repair, regenerate, and generates immune defense response. In fact, RET can be applied in order to improve the prognosis by increasing the length and promoting the root development [ 10 , 14 ] This may also be accepted for improving the prognosis of teeth affected by IRR as this treatment option is able to provide the replacement of missing dental tissues produced by the resorptive lesion [ 4 , 10 ].

Based on the classification of Cveck [ 15 ], our case featured a stage 2 since only half of the root was developed at the moment of the intervention. Following the "clinical considerations to perform a regenerative procedure" given by the American Association of Endodontics, it is indicated to accomplish a RET in order to allow healing and root development [ 16 ].

Taken into consideration that necrotic and infected pulp tissue in IRR must be removed [ 3 , 17 ] and the benefits of intracanal medicaments [ 13 ], calcium hydroxide was used in this case in a two-visit treatment plan. Nevertheless, calcium hydroxide must be used limited in time as it can weaken the fragile root structure and increase the risk of root fracture [ 18 ].

During treatment, mechanical preparation was avoided in order to preserve the already weaken root structure. At the same time, as previously described, a gentle chemical disinfection of the root canal by using 1.5% NaOCl was carried out [ 19 - 20 ]. Though higher NaOCl concentration (e.g. 3% or 6%) should probably have greater antimicrobial capability [ 21 ], at the same time, a higher concentration would be more cytotoxic for periodontal ligament cells and stem cells from apical papilla (SCAPs) responsible for RET [ 20 , 22 ]. However, as elimination of any remainder granulation tissue present in the resorptive lesion and necrotic tissue is essential for the success of the treatment, calcium hydroxide was used as medication between appointments [ 23 ]. The most commonly used medications for RET are based on the combination of three antibiotics (ciprofloxacin, metronidazole, and minocycline) [ 14 ] or two (ciprofloxacin and metronidazole) [ 24 ]. In the present case, calcium hydroxide was chosen as in previous studies [ 25 ] because of its optimal antimicrobial capability to create a basic medium able to neutralize osteoclastic activity [ 26 ] and its ability to promote the proliferation of the stem cells from the dental papilla [ 27 ]. Moreover, according to the European Endodontic Society and the American Association of Endodontists, the use of calcium hydroxide can provide a similar outcome rate as the one produced by an antibiotic paste [ 28 ]. In addition, the possibility of discoloration, bacterial resistance, and allergic reaction generated by the combination of these drugs will be reduced [ 29 ].

The 17% EDTA was used as exclusive irrigant solution during the second appointment in order to remove the calcium hydroxide previously placed and at the same time, promoting environmental conditions that stimulate the survival of SCAPs needed in RET [ 13 , 19 , 20 ] as it stimulate to release growth factors present into the dentin matrix [ 30 ]. Growth factors such as TGF-β1, fibroblast growth factors 2 or vascular endothelial growth factors are essential as they determine the fate of stem cells and help in tissue engineering [ 13 ].

The induction of intra-canal bleeding was carried out with a #30 K file to willfully develop periapical tissue bleeding into the root canal with the aim to provide a blood scaffold. At the same time, platelet-derived growth factors and mesenchymal stem cells will be introduced into the root canal for pulp tissue reparation [ 13 ]. A biocompatible material must be placed between the clot and the final restorative material. Although calcium hydroxide and its derivatives have been described in the literature for this aim, MTA was used in this case as it is one of the most extended material used for this purpose due to its high biocompatibility, essential for the treatment outcome [ 31 ]. 

Two publications reported discoloration after RET in 40% of cases [ 32 - 33 ], as in this case occurred. Although discoloration is more likely to occur with triple antibiotic paste which includes minocycline, it has also been reported with calcium hydroxide and MTA as intracoronal barrier [ 34 ]. In this case, the removal of MTA and a buccal direct composite veneer resulted in a satisfactory aesthetic solution for the patient and tutor. Furthermore, recall examination for at least 36 months is essential for the radiographic assessment of apical healing and root development [ 35 ]. In this case, a follow up to 48 months was carried out.

Nevertheless, some limitations were present in this clinical report and must be taken into consideration. First to mention is the fact that the diagnosis and treatment plan could be compromised in the absence of a CBCT evaluation. Dealing with the individual circumstances of the patients, such as economic reasons, is a reality of the clinician´s routine, as in this case in which a CBCT could not be performed. On the other hand, and at the present time, materials such as Biodentine (BD; Septodont, Saint-Maur-des-Fosses, France) should be of choice because of their improvements over MTA. This clinical case was carried out in 2013, when there was not enough available literature supporting the use of Biodentine and that’s the reason why white MTA was applied. Furthermore, in concordance with the conclusions achieved by Nazzal *et al*. [ 36 ] in a clinical study of traumatized immature teeth with necrotic pulps, this case neither demonstrated continuation of root development. However, apical closure and periapical healing was observed. Given that the patient was a child, the legal guardian signed an informed consent in which the advantages, limitations of this treatment option, consequences of its failure, the alternative therapeutic options, and steps to be followed in case of side effects were noted.

## Conclusion

RET is an alternative modality to treat severely traumatized teeth ongoing the appropriate therapeutic protocols. Future clinical studies should analyze clinical success rate for RET in immature teeth with IRR. 

## Acknowledgement

The authors deny any conflicts of interest related to this study.

## Conflict of Interest

None declared.
